# Clogged Arteries, Safety Net Holes: Treating an Underinsured Patient for Homozygous Familial Hypercholesterolemia With Plasmapheresis

**DOI:** 10.7759/cureus.70174

**Published:** 2024-09-25

**Authors:** Parth Patel, Iheoma Alinnor, Mary Ann Kirkconnell Hall, Lance T Hall, Stacey Watkins

**Affiliations:** 1 Division of Hospital Medicine, Emory University School of Medicine, Atlanta, USA; 2 Hospital Medicine Service, Grady Memorial Hospital, Atlanta, USA; 3 Department of Radiology and Imaging Sciences, Emory University School of Medicine, Atlanta, USA; 4 Nuclear Medicine Service, Grady Memorial Hospital, Atlanta, USA

**Keywords:** apheresis, homozygous familial hypercholesterolemia, low density lipoprotein-cholesterol, low density lipoprotein cholesterol plasmapheresis, therapeutic plasmapheresis

## Abstract

This case report explores the difficulties with rapid lipid-lowering therapies in a resource-limited setting. We present a case of an individual with previously diagnosed homozygous familial hypercholesterolemia presenting with anginal chest pain concerning for non-ST elevation myocardial infarction (NSTEMI), with a low-density lipoprotein (LDL) of 984 mg/dL (reference range: 100-129 mg/dL) and a reversible perfusion defect on his nuclear medicine stress test. In addition to the standard treatment for NSTEMI, including cardiac catheterization, the patient was initiated on a proprotein convertase subtilisin/kexin type 9 inhibitor and underwent two rounds of plasmapheresis, which effectively and rapidly lowered his LDL levels.

## Introduction

Familial hypercholesterolemia (FH) results from either heterozygous or homozygous mutations in the low-density lipoprotein receptor (LDLR), apolipoprotein B (APOB), or proprotein convertase subtilisin/kexin type 9 (PCSK9) genes, leading to significantly elevated low-density lipoprotein cholesterol (LDL-C) levels, generally in a pattern of autosomal dominance. FH is associated with greatly elevated LDL-C levels and increased risk of morbidity and mortality associated with cardiovascular disease, particularly atherosclerotic cardiovascular disease [1]. Individuals who are homozygous for a specific mutation have more severe, earlier onset homozygous FH (HoFH) that is often apparent in childhood. Heterozygous FH affects 1 in 200-250 people globally [2], or 35 million people worldwide [3]; HoFH is rarer, estimated at 1 in 170,000-300,000 [4]. The diagnosis of HoFH is usually based on untreated LDL-C >500 and the presence of cutaneous xanthomas before 10 years of age [5,6].

This case report explores difficulties with rapid lipid-lowering therapies in a resource-limited setting. An individual with previously diagnosed FH that worsened significantly when he lost insurance and access to medications presented with chest pain suggestive of non-ST elevation myocardial infarction (NSTEMI), with an LDL-C of 984 mg/dL and a reversible perfusion defect on his nuclear medicine stress test. In addition to standard NSTEMI treatment, he received a proprotein convertase subtilisin/kexin type 9 (PCSK9) inhibitor and underwent two rounds of plasmapheresis, effectively and rapidly lowering LDL-C levels.

## Case presentation

The patient was a 40-year-old man; originally from Nigeria, he immigrated to the United States nine years before presentation. He presented with typical anginal pain, including sharp, left-sided pain that was worse with exertion and improved with rest and dyspnea on exertion. In the emergency department, he had elevated blood pressure on initial assessment. His initial high-sensitivity troponin was elevated and then trended down; other significant labs are listed in Table 1. An electrocardiogram found no acute ischemic changes.

**Table 1:** Initial laboratory values

Laboratory assay	Value	Reference range
High-sensitivity troponin	579 ng/L	<20 ng/L
Brain natriuretic protein	118 pg/mL	<78 pg/mL
Total serum cholesterol	1034 mg/dL	170-239 mg/dL
Low-density lipoprotein cholesterol	984 mg/dL	100-129 mg/dL
Blood urea nitrogen (BUN)	6 mg/dL	8-23 mg/dL
Creatinine	0.8 mg/dL	0.7-1.3 mg/dL
BUN/creatinine ratio	7.5:1	10:1-20:1

**Figure 1:**
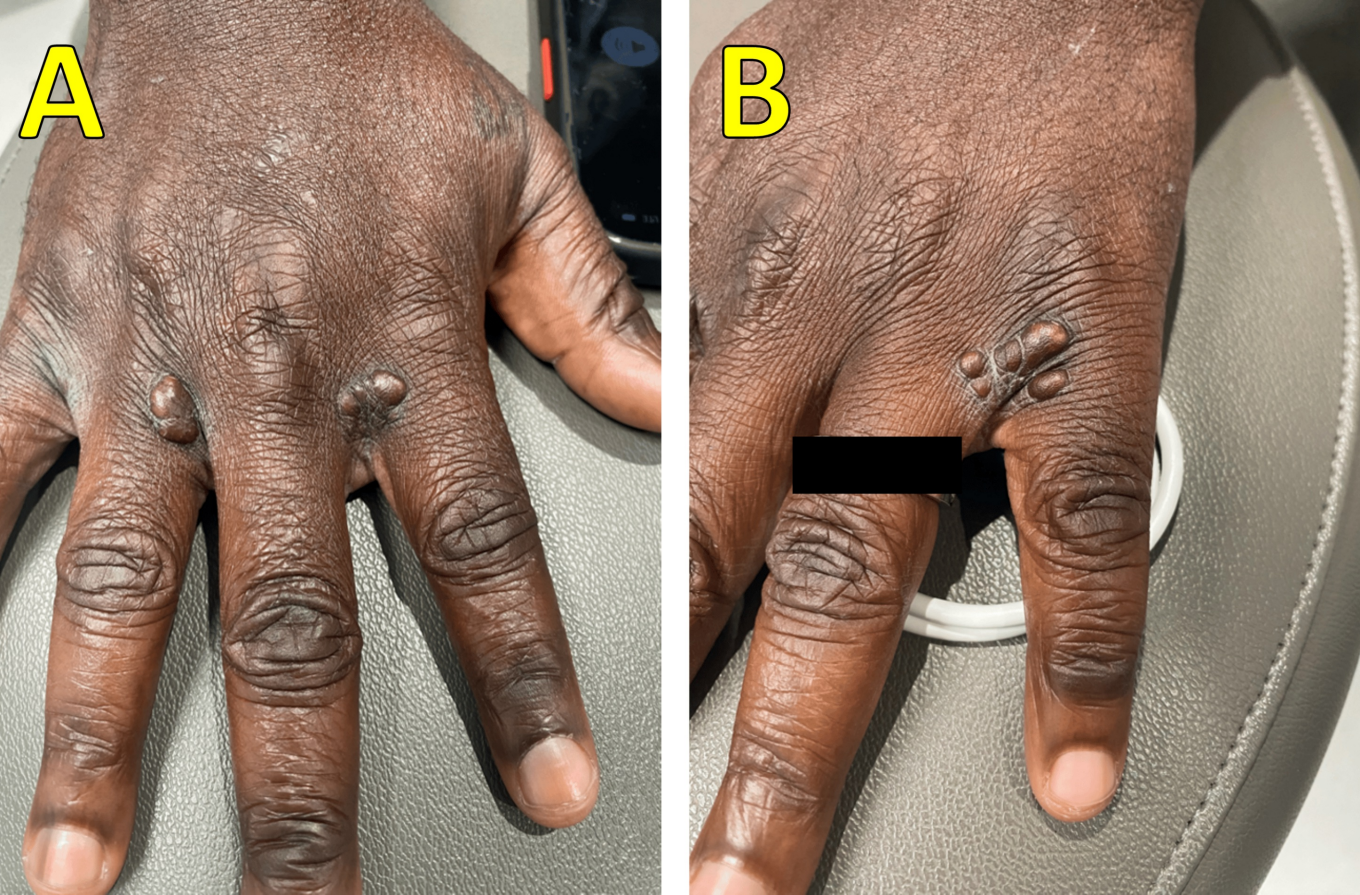
Physical exam images: right hand (A) and left hand (B) with planar xanthomas from the accumulation of lipids in the dermis Xanthomas found in the webspace between the fingers (and toes) are pathognomonic for familial hypercholesterolemia.

**Figure 2:**
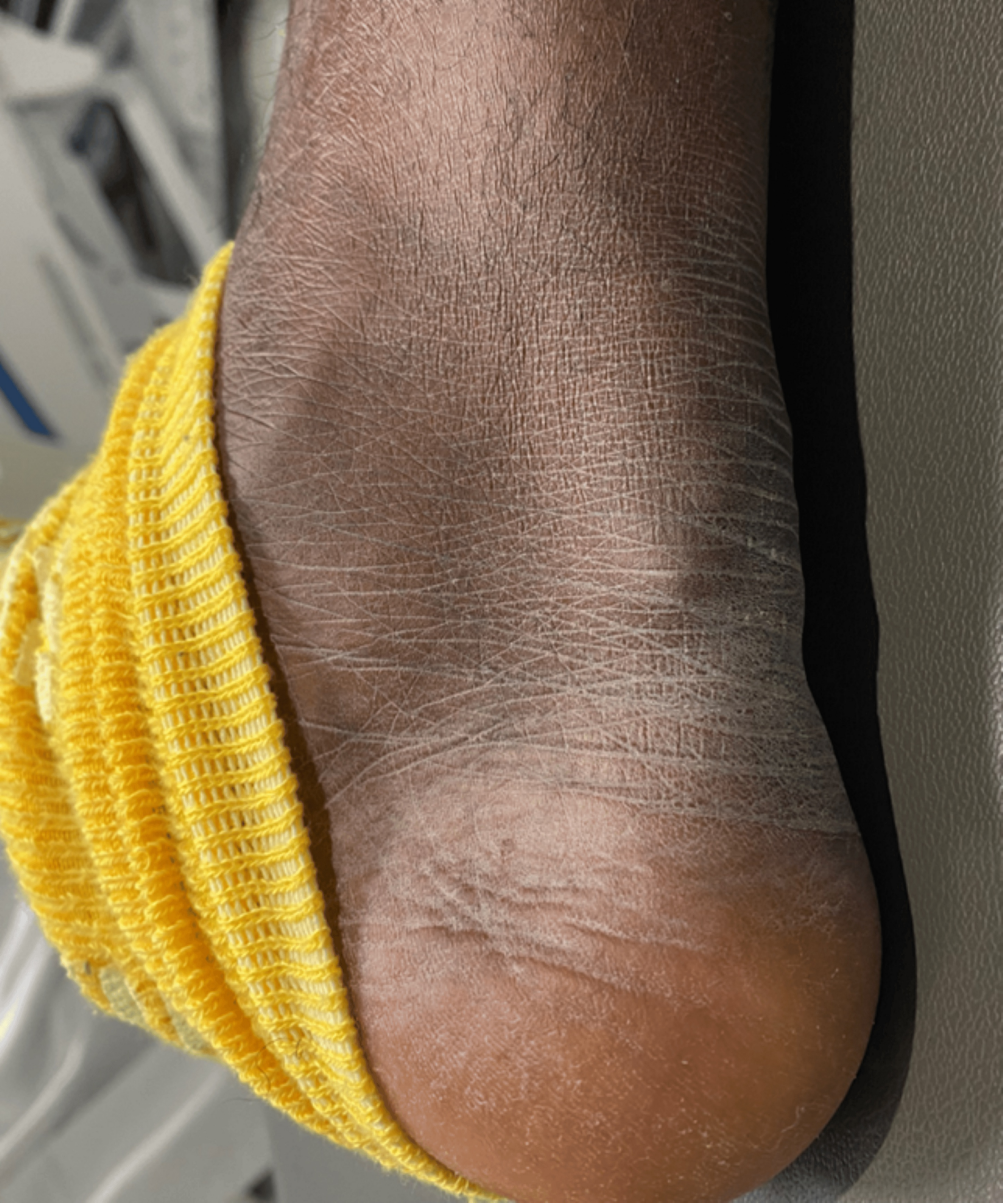
Xanthoma in the right Achilles tendon/heel This xanthoma developed due to the deposition of lipids within the tendons. The Achilles tendon is one of the most common locations for this to occur, and typically, the overlying skin color is not abnormal for the patient.

**Figure 3:**
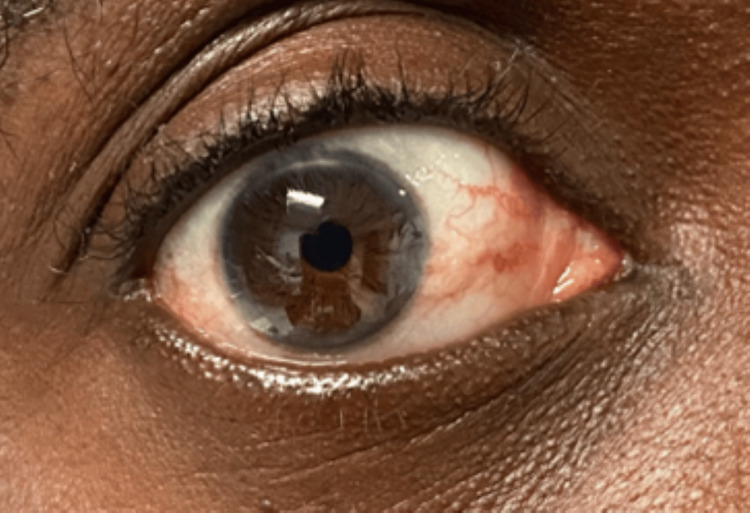
Physical exam image: right corneal arcus Arcus is caused when lipids deposit within the peripheral cornea. While this may be seen normally in elderly individuals, this finding is specific to familial hypercholesterolemia in younger individuals.

**Figure 4:**
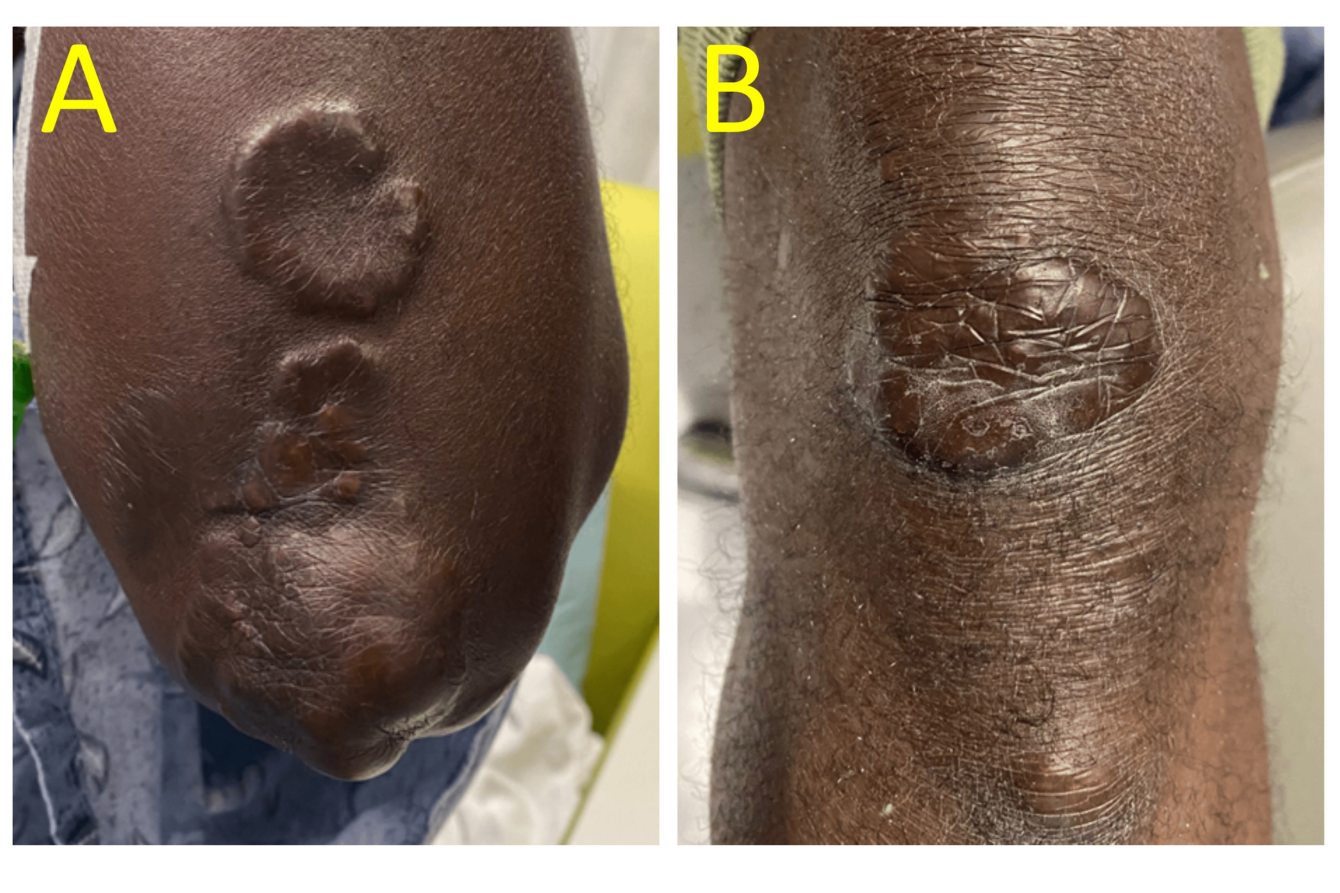
Physical exam image Left elbow (A) and right knee (B) with the early development of tuberous xanthomas: firm, painless nodules that typically develop over areas under pressure, including the knees and elbows.

He exhibited several classic physical exam findings for HoFH-related cholesterol deposition, including corneal arcus, and tendinous xanthomas of the bilateral Achilles, knees, elbows, and finger joints (Figures 1-4).

His history provided important clues. As a child, he had noticed flesh-colored papules on the extensor surfaces of his knees and elbows and smaller papules in the dorsal interdigital spaces of his fingers. He reported a significant family history of cardiac disease and early mortality; his mother, who had similar physical changes, died of a stroke at age 60, his father of heart problems at age 79, and one sister of presumed sudden cardiac death at age 26.

He noticed progressively worsening dyspnea on exertion in his early 30s, particularly when walking with friends of the same age. When he came to the United States of America at age 31, he had his dyspnea evaluated by a physician who found a highly elevated lipid panel. He underwent extensive cardiac workup and received a coronary artery bypass graft (CABG) at age 32. Since his CABG, he had been followed by cardiologists with a regimen of statin, ezetimibe, and niacin.

He had moved to Atlanta two years earlier, establishing care with a local cardiologist who did not change his medication regimen. Unfortunately, he lost his job and, subsequently, his health insurance coverage in March 2023. Between March and July, he could not obtain or take his prescribed cholesterol-lowering medications.

The differential diagnosis for chest pain in an individual with known HoFH is limited. Of greatest concern was acute coronary syndrome given his known accelerated premature atherosclerotic cardiovascular disease. Alternatively, aortic stenosis is an additional reported cardiovascular complication of HoFH that could also contribute to angina [7]. Lastly, rather than anchoring solely on the known cardiovascular complications associated with HoFH, one should consider more common etiologies for chest pain, including pulmonary embolism, aortic dissection, pericarditis, and congestive heart failure exacerbation. Given his clinical presentation in the setting of lack of access to essential medication, the working diagnosis of accelerated premature atherosclerotic cardiovascular disease was established.

**Figure 5:**
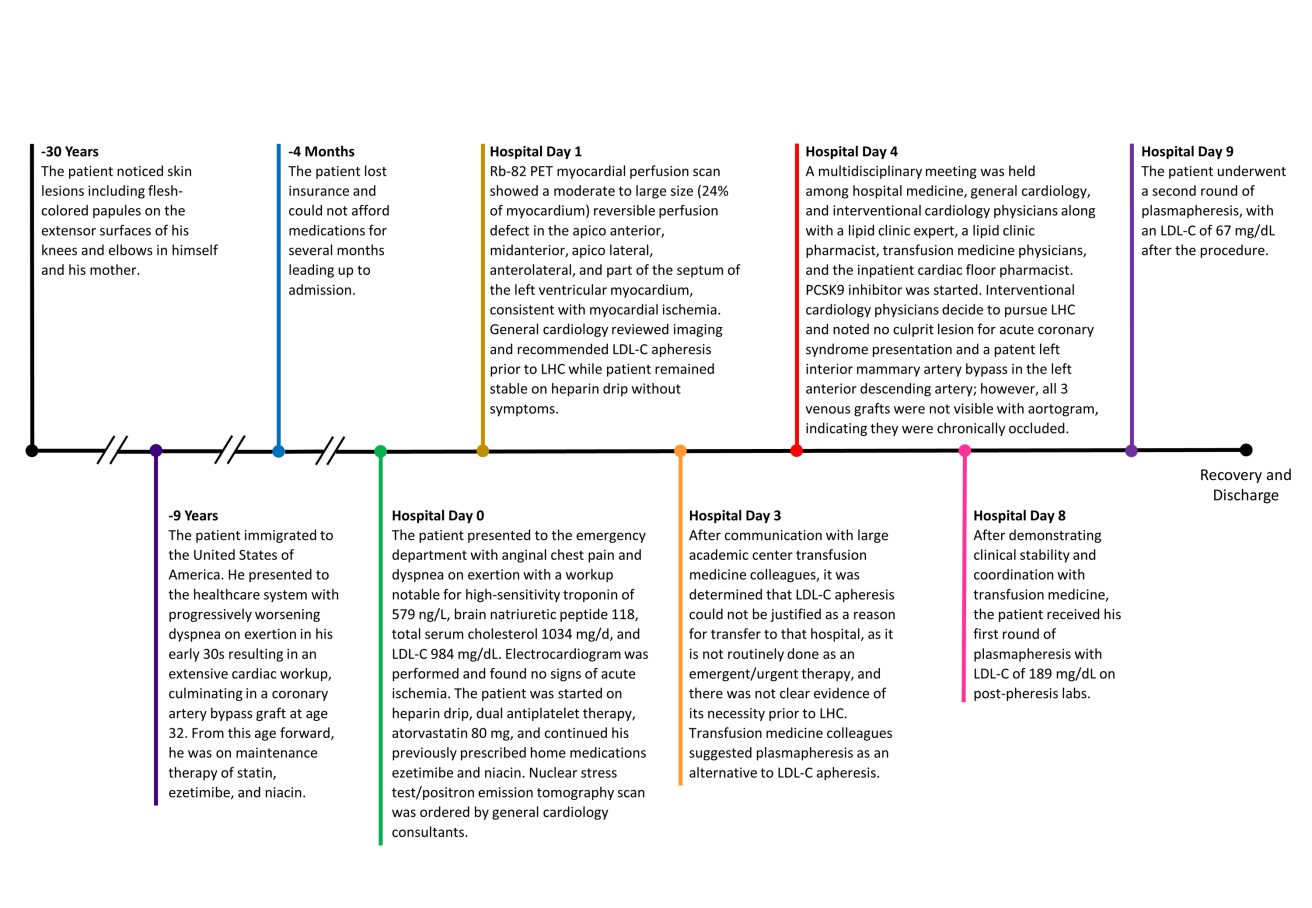
Case timeline

We had immediate concerns about NSTEMI given the history of CABG and new anginal chest pain, so general cardiology was consulted. He was started on a heparin drip and dual antiplatelet therapy and restarted on oral lipid-lowering medications (see Figure 5 for a comprehensive clinical timeline). He was admitted to the hospital medicine service for further workup.

**Figure 6:**
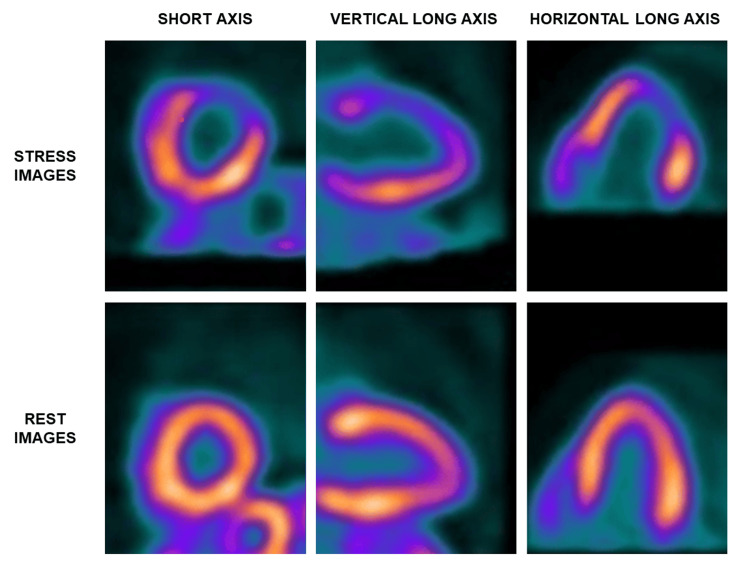
Rb-82 PET myocardial perfusion scan selected slices The Rb-82 PET myocardial perfusion scan selected slices with stress images (top row) and rest images (bottom row) in the short axis (left column), vertical long axis (middle column), and horizontal long axis (right column) views, respectively. Stress images demonstrate a moderate to large size perfusion defect with severely reduced to absent counts in the apico to mid-anterior, apicolateral, anterolateral, and extending to the septum, which reverses on rest images. This is consistent with significant myocardial ischemia.

**Figure 7:**
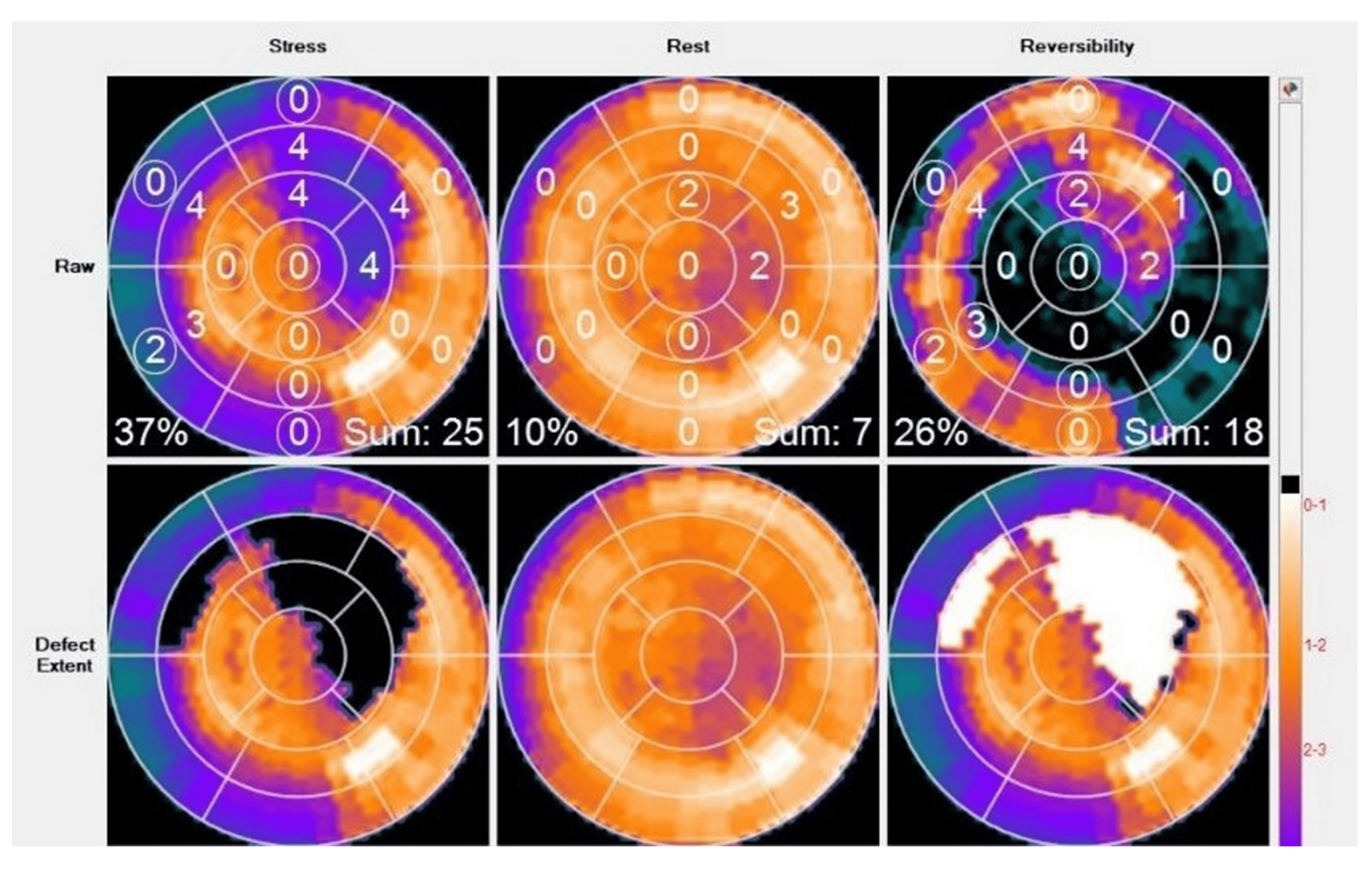
Polar maps of an Rb-82 PET myocardial perfusion scan with stress (left column), rest (middle column), and reversibility (right column) maps The stress map demonstrates a moderate-to-large sized perfusion defect with severely reduced to absent counts (dark defects) in the apico to mid-anterior, apicolateral, anterolateral, and extending to the septum which near-completely reverses on rest images. The reversibility map displays the reversible defect (best seen in the bottom right map in white).

An Rb-82 positron emission tomography (PET) myocardial perfusion scan performed shortly after he was admitted showed a moderate- to large-size (24% of the myocardium) reversible perfusion defect in the apico-anterior, mid-anterior, apico lateral, anterolateral, and part of the septum of the left ventricular myocardium, consistent with myocardial ischemia (Figure 6 and Figure 7).

The general cardiologist recommended lipid reduction before diagnostic and potentially therapeutic left heart catheterization (LHC), strongly advising LDL-C apheresis to rapidly reduce LDL levels prior to catheterization to optimize for percutaneous coronary intervention (PCI).

However, LDL-C apheresis was available at only one center in the city. Given the lack of strong data related to the rapid lowering of LDL in acute coronary syndrome in HoFH patients, there was no justification for patient transfer; his lack of insurance coverage and the associated costs factored into this decision. We consulted transfusion medicine colleagues who suggested an alternative and acceptable option: plasmapheresis, which is readily available at more sites (including ours) and has a class 2 indication for lipid lowering.

A multidisciplinary team discussed the case, including hospital medicine, general cardiology, interventional cardiology, lipid clinic and transfusion medicine physicians, and lipid clinic and inpatient cardiology pharmacists. Interventional cardiology agreed to perform LHC, despite the patient’s elevated LDL-C levels, followed by plasmapheresis and initiation of a PCSK9 inhibitor, alirocumab.

Interventional cardiology performed diagnostic LHC with no evidence of an acute culprit causing acute coronary syndrome and noted a patent left internal mammary artery to the left anterior descending artery; however, none of the three venous grafts were visible with an aortogram, indicating chronic occlusion. Cardiothoracic surgery evaluated the LHC images and recommended no acute intervention and no role for surgery, with consideration of complex PCI, which was not pursued.

Upon administration of oral medications (80 mg atorvastatin, ezetimibe, niacin) and a newly initiated PCKS9 inhibitor, the patient’s LDL-C decreased to 786 mg/dL. He then had a temporary central venous catheter placed for plasmapheresis. After the first plasmapheresis session, LDL-C was dramatically lowered to 189 mg/dL, and after the second session, it declined to 67 mg/dL.

The patient was discharged in stable condition and was scheduled for a clinic visit 30 days after his first subcutaneous dose of alirocumab. Our institution’s pharmacy is assisting with obtaining continued alirocumab therapy through the pharmaceutical manufacturer’s patient assistance program, given his lack of health insurance.

## Discussion

HoFH is extremely rare, with little data on management in acute settings. Our patient reported being managed on oral medications alone while he had insurance; he had never been on a PCSK9i nor undergone any plasma exchange therapy. Losing access to care and medications likely precipitated his symptoms and potentially accelerated the chronic graft occlusions noted in LHC. Several prior reports have detailed appropriate treatment regimens as well as algorithms for HoFH in the outpatient setting but our case highlights an acute presentation warranting immediate inpatient interventions [8-10].

FH treatment centers on the maximal lowering of LDL-C levels. The backbone of pharmacotherapy is statins, which even at high doses only deliver moderate reductions (10-25%) in LDL-C in most FH patients and are often coupled with ezetimibe (an additional 10-15% reduction) [5,9]. Other cholesterol-lowering medications, such as PCSK9 inhibitors, bile acid sequestrants, niacin, and fibrates, are also used. Oftentimes, despite maximal therapy, there is a <50% reduction in LDL-C or residual severe elevations of LDL >300 mg/dl or >200 mg/dL with prevalent cardiovascular disease [5]. In such cases, LDL apheresis, an extracorporeal treatment removing apoB-containing lipoproteins from plasma, can be entertained. However, this modality is not widely available or accessible.

In addition to his social situation, the rarity of our patient’s condition meant that some therapies recommended and pursued were not based on strong evidence-based data. LDL-C apheresis, for example, is oftentimes only seen in an outpatient setting as monthly maintenance therapy for chronic stabilization. In this case, LDL-C apheresis was recommended as an acute lipid-lowering treatment, a novel therapy in the inpatient setting. However, LDL-C apheresis is a scarce resource only available at highly specialized centers. Plasmapheresis is not regularly used for lipid-lowering; although we did see remarkable levels of effectiveness with this intervention, the short- or long-term benefits of acute lipid lowering for atherosclerotic cardiovascular disease risk or acute management of cardiac disease remain unclear.

## Conclusions

Treatment interruptions are suboptimal for any illness or condition. While cessation of some medications will lead to immediate morbidity or mortality, interruptions to others can cause significant delayed and less readily apparent (but no less potentially fatal) harm to patients. Treatment interruption in patients with HoFH can be life-threatening, which highlights the need for easy access to lipid-lowering agents. Given the cost and lack of access to newer medications and therapies, including LDL-C apheresis, healthcare providers should consider plasmapheresis in the acute setting to rapidly lower lipid levels.
